# Heritability and Genetics of Type 2 Diabetes Mellitus in Sub-Saharan Africa: A Systematic Review and Meta-Analysis

**DOI:** 10.1155/2020/3198671

**Published:** 2020-06-30

**Authors:** Evans Adu Asamoah, Christian Obirikorang, Emmanuel Acheampong, Max Efui Annani-Akollor, Edwin Ferguson Laing, Eddie-Williams Owiredu, Enoch Odame Anto

**Affiliations:** Department of Molecular Medicine, School of Medicine and Dentistry, Kwame Nkrumah University of Science and Technology, Ghana

## Abstract

**Objectives:**

Sub-Saharan Africa (SSA) is observing an accelerating prevalence rate of type 2 diabetes mellitus (T2DM) influenced by gene-environment interaction of modifiable and nonmodifiable factors. We conducted a systematic review and meta-analysis on the heritability and genetic risk of T2DM in SSA.

**Methods:**

We reviewed all published articles on T2DM in SSA between January 2000 and December 2019 and available in PubMed, Scopus, and Web of Science. Studies that reported on the genetics and/or heritability of T2DM or indicators of glycaemia were included. Data extracted included the study design, records of family history, pattern and characteristics of inheritance, genetic determinants, and effects estimates.

**Results:**

The pattern and characteristics of T2DM heritability in SSA are preference for maternal aggregation, higher among first degree compared to second-degree relatives; early age-onset (<50 years), and inherited abnormalities of beta-cell function/mass. The overall prevalence of T2DM was 28.2% for the population with a positive family history (PFH) and 11.2% for the population with negative family history (NFH). The pooled odds ratio of the impact of PFH on T2DM was 3.29 (95% CI: 2.40-4.52). Overall, 28 polymorphisms in 17 genes have been investigated in relation with T2DM in SSA. Almost all studies used the candidate gene approach with most (45.8%) of genetic studies published between 2011 and 2015. Polymorphisms in *ABCC8*, *Haptoglobin*, *KCNJ11*, *ACDC*, *ENPP1*, *TNF-α*, and *TCF7L2* were found to be associated with T2DM, with overlapping effect on specific cardiometabolic traits. Genome-wide studies identified ancestry-specific signals (*AGMO-rs73284431*, *VT11A-rs17746147*, and *ZRANB3*) and *TCF7L2-rs7903146* as the only transferable genetic risk variants to SSA population. *TCF7L2-rs7903146* polymorphism was investigated in multiple studies with consistent effects and low-moderate statistical heterogeneity. Effect sizes were modestly strong [odds ratio = 6.17 (95% CI: 2.03-18.81), codominant model; 2.27 (95% CI: 1.50-3.44), additive model; 1.75 (95% CI: 1.18-2.59), recessive model]. Current evidence on the heritability and genetic markers of T2DM in SSA populations is limited and largely insufficient to reliably inform the genetic architecture of T2DM across SSA regions.

## 1. Introduction

Studies on chronic noncommunicable diseases (NCDs) have gained considerable attention in Africa. This is as a result of the dramatic shift in the profile of diseases burden with the dominance of communicable diseases to NCDs in Sub-Saharan Africa (SSA) [1]. Africa constitutes the highest proportion of people with undiagnosed diabetes (>50%) and has been projected to experience further future increase (>100%) in the burden of diabetes by 2045 [2]. Between the periods of 1990 and 2017, NCDs have accounted for a 67% increase in disability-adjusted life-years (DALY) in SSA, and type-2 diabetes mellitus (T2DM) accounted for 126% increase in DALY [3]. The regional prevalence of T2DM in SSA is reportedly 5.1% [4], which range from 2.6% to 22.5% across data from different regions in SSA [5]. Between 1990 and 2015, T2DM has accounted for 7% mortality rate attributed to NCDs in SSA [6].

In Africa, ageing and globalisation including nutrition transition and adoption of sedentary lifestyles are reportedly key factors accelerating diabetes prevalence, in line with the worldwide rise in overweight and obesity [7, 8]. The causal linkages of T2DM reported in most studies in Africa include environmental/behavioural cause, direct multifactorial cause, and indirect multifactorial cause, respectively. Direct multifactorial cause involves a network of risk factors directly causes T2DM overtime while indirect multifactorial cause is where aggregation of risk factors over a period cause a precursor health condition (obesity, hypertension, metabolic syndrome, etc.) that leads to T2DM [9]. Accumulated evidence from a number of studies confirms a gene-environment interaction of modifiable and nonmodifiable factors, which promote the development of diabetes [10–12]. However, a limitation to the understanding of the epidemiology of T2DM in SSA has been the lack of established vital statistics systems and reliable population-level data for most countries [13]. More unfavourably, in the face of recent implementations of high throughput genotyping and sequencing approaches to advance recent understanding of the genetic basis of T2DM, countries in SSA have not been adequately represented in the global genomic efforts [14]. Thus, studies to provide ample data in the areas of heritability, family history, and genetic risk of T2DM are warranted. As such, these studies will highlight new genetic initiatives towards the prevention and treatment of T2DM in SSA.

T2DM phenotype in SSA has been characterized by early onset in a younger population (<50 years) [15] and pancreatic beta-cell secretory dysfunction which manifests as the blunted acute first phase of insulin secretion rather than peripheral insulin resistance [16, 17]. These characteristics of T2DM have been confirmed in the reports of Oli et al. [18] and Bakari and Onyemelukwe [19]. Thus, T2DM in SSA like the Asians [20], but unlike the Europeans [21], follows a different course that reflects environmental and genetics contribution to the high burden. Whereas the contribution of environmental factors to the development of T2DM in SSA is well established, the contribution of genetic factors remains elusive. There is currently no thorough systematic review and meta-analysis reporting on the genetics and heritability of T2DM in SSA. An up-to-date and accurate documentation on the genetics and heritability of T2DM in SSA is needed to highlight gaps and if applicable inform policymakers to design and implement public health intervention programs. This study recapitulates literature on the genetics and heritability of T2DM currently available in SSA.

## 2. Methodology

### 2.1. Search Strategy and Selection Criteria

Following the Preferred Reporting Items for Systematic Reviews and Meta-Analyses (PRISMA) guidelines [22] and the protocol for conducting a systematic review for genetic association studies [23], we conducted a systematic review of all papers published on T2DM in Sub-Saharan Africa between January 2000 and November 2019 and available on PubMed, Scopus, and Web of Science. We defined Sub-Saharan Africa as all mainland African countries south of the Sahara excluding Algeria, Djibouti, Egypt, Libya, Morocco, Somalia, Sudan, and Tunisia. Boolean logic with search terms including “type 2 diabetes”, “non-insulin-dependent diabetes mellitus”, Type-2 diabetes mellitus”, and “Africa south of Sahara” were used. We applied the Medical Subject Heading terms (MeSH) to identify synonyms. We limited our search to studies done in English. The study protocol and detailed search parameters are attached to this manuscript as Supplementary Material (available here). The reference lists of study reports and review articles were scanned to identify additional articles validated in Science Citation Index Expanded, published on and after January 2000.

Two authors (EAA and EA) independently reviewed the title and abstracts of studies for inclusion. Cross-sectional studies (community-based, population-based, hospital-based, and comparative), case-control, and prospective cohort studies that reported a heritability, family history, and genetic risk of T2DM in SSA were eligible for inclusion. We regarded the following terms as equivalent to type-2 diabetes mellitus: “Type 2 Diabetes”, “Diabetes Mellitus, Slow Onset”, “Noninsulin-Dependent Diabetes Mellitus”, “Maturity Onset Diabetes Mellitus”, “Diabetes Mellitus, Stable”, “Diabetes Mellitus, Type II”, “Adult-Onset Diabetes Mellitus”, “Diabetes Mellitus, Noninsulin Dependent”, “NIDDM”, “Diabetes, Maturity-Onset”, “Diabetes, Type 2”, “Slow-Onset Diabetes Mellitus”, “Diabetes Mellitus, Non-Insulin-Dependent”, “Diabetes Mellitus, Ketosis-Resistant”, “Ketosis-Resistant Diabetes Mellitus”, and “Maturity-Onset Diabetes Mellitus”. Two investigators (EAA and EWO) did a second review to assess the entire articles and their reference list. Any disagreement resulted in a joint review of the article with reconciliation. Details of inclusion and exclusion criteria can be found in Supplementary Appendix, pp 4.

### 2.2. Data Extraction

After identifying articles for inclusion, two authors (EA and CO) independently reviewed each article for data extraction into a standard, preformulated form:

*Family History of T2DM*: data were extracted under the following headings: name of first author, publication year, Sub-Saharan Africa region, country, ethnicity, study design, participants, sampling technique, and sample size, criteria for T2DM definition, number of participants with a positive family history (PFH), T2DM prevalence in PFH, and T2DM prevalence in negative family history (NFH).

*Heritability*: themes were used to pool key concepts and interpreted within and across studies. Themes used included T2DM aggregation among families, onset of T2DM among probands, glucose tolerance, and effectiveness mechanisms among probands and control participants.

*Genetic Risk*: data were extracted under the headings—first author and publication year; the population; gene (s) and allelic variant (s); total sample size; the number of nucleotide genes in which they are found and their corresponding genotypes both in cases and controls, genotyping method; minor allele frequency.

### 2.3. Statistical Analysis

For the polymorphisms investigated in multiple studies, we derived the pooled estimates of their association with T2DM risk across studies using a random effect model meta-analysis, implemented using MedCalc Software for Windows, version 18.91 (https://www.medcalc.org/). Heterogeneityin the measure of association across studies was further quantified with the *I*^2^ statistic, with value < 25% indicating low heterogeneity, 25–50% indicating moderate heterogeneity, 50–75% indicating high heterogeneity, and>75% indicating extreme heterogeneity [24]. Publication bias was assessed using funnel plots [25].

## 3. Results

### 3.1. Literature Search

**Figure 1:**
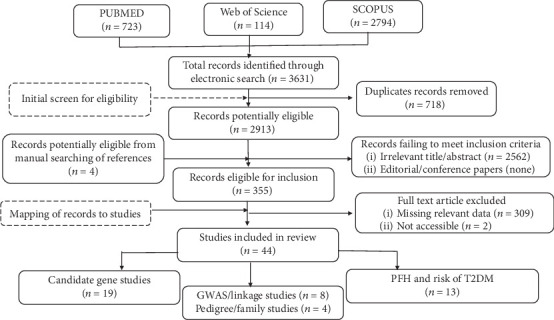
Flowchart for the study selection process.

The search strategy and the number of hints per each search are shown in Supplementary Appendix pp 1-2. A total of 3,519 published records were identified through searches (Figure 1). A total of 718 studies were excluded based on duplication, and titles/abstracts of 2913 records were screened. Of these, 341 were included for full-text review, and 4 additional studies were obtained from screening the references of included studies. A total of 44 studies consisting of 8 GWAS/linkage studies, 4 pedigree/familial studies, 19 candidate gene association studies, and 13 population/hospital-based cross-sectional studies were included. Figure S1 shows the linear growth over time of heritability, family history, and genetic studies on T2DM published in SSA. Up to 45.8% of genetic studies were published between 2011 and 2015, and 66.7% of FH and heritability were published between 2011 and 2019.

### 3.2. Pedigree and Family Studies on T2DM in SSA

Although few published studies [26–29] have thoroughly investigated the heritability of T2DM within the SSA context, these studies demonstrate a distinct familial aggregation of T2DM and offered useful insights into the pattern of heritability of T2DM.

The reports of Meiloud et al. [26] among 609 known T2DM Mauritanian patients indicated that 27% of T2DM patients have at least one relative with the condition. The association between PFH and T2DM was observed to be higher among first-degree compared to second-degree relatives (*p* = 0.003). Moreover, more probands with an affected mother than those with affected father were observed, suggesting a preferential maternal effect which does not extend to second-degree relatives.

Similar to the above findings, a prospective case-control study among 1,111 T2DM and 687 controls from the Black South African population [27] reported 27.3% of T2DM having a PFH compared with 8.4% in the control group (*p* < 0.01). Also, first-degree relatives with T2DM had a significant maternal aggregation of 64.7% compared with 27% of those who had diabetic fathers (*p* < 0.01). In addition to these, Erasmus et al. [27] reported that patients with PFH had an earlier onset (<50 years) of diabetes than those with NFH (*p* < 0.01).

Two of the studies included in this review reported severe pancreatic beta-cell secretory dysfunction among Ghanaian and Cameroonian offspring of T2DM patients compared with those of nondiabetic parents [28, 29]. Amoah et al. [29] compared *β*-cell secretion, insulin secretion, insulin sensitivity (Si), and glucose effectiveness (Sg) among 42 healthy nondiabetic first-degree relatives and 22 healthy control subjects without a PFH The mean total and acute first and second phases of serum insulin and c-peptide responses after oral glucose tolerance test at *t* = 60, 90, and 120 minutes (*p* < 0.05) were prolonged in relatives than in healthy controls. The level of insulin resistance and glucose effectiveness at basal insulin level (Sg) did not significantly differ in the relatives and healthy controls [29].

Even though only few studies (<5) have investigated the familial aggregation of T2DM in SSA, three common themes were identified to be associated with T2DM heritability. Firstly, preference for maternal aggregation was higher among first degree compared to second-degree relatives; secondly, probands manifested a degree of *β*-cell impairment marked by reduced early-phase insulin secretion; and lastly, T2DM onset was early (<50 years) among relatives of diabetic individuals.

### 3.3. Genome-Wide Association/Linkage Studies

The two maiden genome-wide linkage study in SSA among the Akan and Ga, from Ghana, Yoruba, and Igbo from Nigeria identified significant linkage signal regions on 10q23 and 4p15, while few other regions had logarithm odd (LOD) scores considered as suggestive evidence of linkage [30, 31].

Using an affected sibling pair (ASP) approach with 390 short tandem repeat markers typed in 343 families in the African American Diabetes Mellitus (AADM) study, a multipoint linkage analysis identified suggestive evidence of linkage in four regions on three chromosomes; 12 (LOD = 1.92), 19 (LOD = 1.81), and 20 (LOD = 2.63), with the strongest evidence on 20q13.3 (LOD = 1.80). This region was previously identified as a T2DM susceptible locus in a non-African population [32, 33]. In addition, 12q24 linkage signal was identified, as has been previously reported [34] as T2DM susceptible locus.

Chen et al. [31] employed the multipoint linkage approach performed on log C-peptide using the AADM samples. Significant linkage signals were observed on 10q23 (LOD = 4.04), 4p15 (LOD = 3.48), 15q14 (LOD = 2.41), and 18p11 (LOD = 2.18). These regions were identified to harbour five positional candidate genes for T2DM and related complications: the pituitary adenylate cyclase-activating polypeptide (*PACAP*) in 18p11; the peroxisome proliferator-activated receptor-gamma coactivator 1 (*PPARGC1*) in 4p15; *PTEN*, *PPP1R5*, and *IDE* in 10q23 (Supplementary Appendix, pp.16).

Adeyemo et al. [35] conducted a study on the evaluation of T2DM susceptibility genome-wide loci in SSA. The study sampled 1775 subjects of which 90% were participants enrolled in the AADM study and 10% of East Africans enrolled from Kenya. The study observed 41 loci showing transferability to African sample: 11 at the exact reported SNP and 30 others at SNPs in linkage disequilibrium (LD) with the reported SNP. The *TCF7L2* SNP rs7903146 showed the strongest association with T2DM (*p* = 1.61 × 10^−8^, OR 1.50, 95% CI 1.26–2.15) [35]. When the 41 loci that showed transferability were fine-mapped, stronger evidence of association with T2DM was shown by neighbouring SNPs (*SLC30A8* and *CDKAL1*) compared with index SNPs with an exception to the block that contained both *TCF7L2* and *ZBED3*.

In a recent discovery study by Adeyemo et al. [36] using the population group of West and East Africans [35], *ZRANB3* (lead SNP *p* = 2.831 × 10^−9^), which has not been previously associated with T2DM, was identified as an African-specific T2DM locus. The role of *ZRANB3* in an experimental model was found to be involved in *β*-cell functional response to high glucose conditions, thus the capacity of the pancreas to respond to insulinogenic stimuli [35].

A study by Chen et al. [37] found an association with T2DM using the shared variant (rs7903146) from West, East, and South Africa, the Europeans, and Africans and a distinct African-specific signal, *VT11A- rs17746147*. Likewise, one novel signal, rs73284431, near alkylglycerol monooxygenase (*AGMO*: *p* = 5.2 × 10^−9^, MAF = 0.095), distinct from previously reported signals in the African region was also detected. This indicates that combining different populations from SSA could possibly lead to the discovery of new and population-specific loci for T2DM.

### 3.4. Association between PFH and the Risk of T2DM among SSA Population

Thirteen studies were identified that provided evidence on the risk of T2DM among the general population. Ten of the studies were population/community-based cross-sectional studies [38–47], two were hospital-based case-control studies [48, 49], and one study was a comparative cross-sectional study [50]. A total of 14,432 participants from the ten cross-sectional studies and the two hospital-based case-control studies were used for meta-analysis. The characteristics of the 12 studies are shown in Table S4 (Supplementary Appendix pp. 10). From the pooled studies, the prevalence of T2DM ranged from 5.4-66.8% among participants with a positive family history (PFH) and 2.5-32.2% among participants with no family history (NFH). The overall prevalence of T2DM among the SSA population with PFH of T2DM was 28.2% compared with 11.2% among participants with NFH (Table 1).

**Figure 2:**
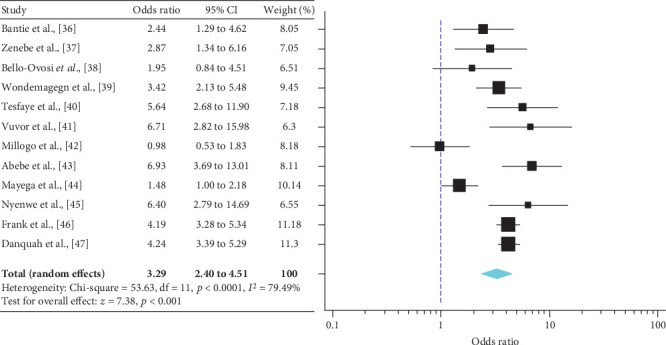
Forest plots for meta-analysis of family history and the risk of T2DM among SSA population.

**Figure 3:**
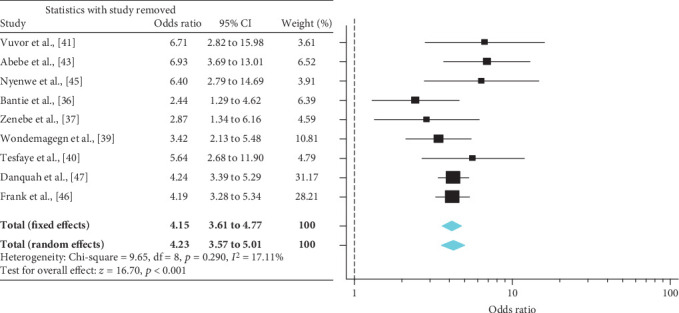
Leave-one-out sensitivity analysis of the effect of PFH on the risk of T2DM.

**Figure 4:**
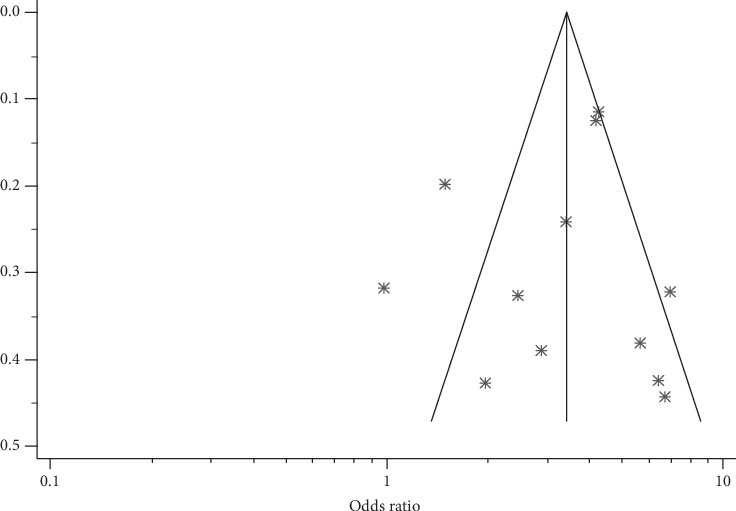
Funnel plots of studies reporting on family history and the risk of T2DM in SSA.

A meta-analysis from the twelve studies demonstrated a significant effect of PFH on T2DM prevalence in SSA [OR = 3.29 (95% CI: 2.40-4.52)] (Figure 2). This effect size was robust in the sensitivity analysis and remained at a significant level following the omission of each single study. A leave-one-out sensitivity analysis also revealed that the pooled estimate was much less impacted by odds ratios values from Bello-Ovosi et al. [40], Millogo et al. [44], and Mayega et al. [46] (Figure 3). The funnel plot revealed publication bias, depicted by the asymmetrical display of odds ratios reported by the various studies (Figure 4).

### 3.5. Candidate Gene Studies

A total of 28 polymorphisms (including SNP, indels, and repeats) in 17 genes were investigated across studies. Supplementary Document: pp. 6-10 shows the distribution of the polymorphisms in various genes, including four in *CAPN10* (rs3792267, rs2975762, rs5030952, rs3842570), three in *TCF7L2* (rs7903146; rs12255372; DG10S478), and *FTO* (rs9941349, rs3751812, rs8050136); and two in *ACDC* (C-11377G, G-11391A) and *ENPP1* (rs997509, rs1044498).

A shown in Supplementary Table S3, 8 SNPs in the *SCL40A1*, *PPAR-γ*, *IRS-1*, *AGRP*, *PPAR-α*, and *CAPN10* genes were examined and were not significantly associated with T2DM or measures of glycaemia across included studies. In single studies, the *ABCC8* and Haptoglobin SNPs were found to be associated with T2DM among Nigerians [51] and Ghanaians [52], respectively. Two polymorphisms in two genes (*PSMD6* and *C2D4B*) were found to be nonsignificantly associated with T2DM in Black South Africans after correcting for multiple testing [53]. Polymorphisms in *KCNJ11 (rs5219)* was found to be nonsignificantly associated with T2DM among Ghanaian [49] and Nigerian [54] populations but significant among Mauritanians [55].

Of the three polymorphisms in the *FTO* gene reported in two studies in two black South African mixed ancestry population, only *rs9941349* (OR = 1.43 (1.00-2.04), *p* = 0.052) was near significance, associated with T2DM after adjustment for cofounders [56].

With the two SNPs in *ACDC* reported among Black South Africans, *SNP G-11391-A* was found to be protective to T2DM [57]. Of the two SNPs in *ENPP1* reported among South-African Mixed ancestry, only *rs997509* was found to be significantly associated with T2DM [56]. Also, one SNP in TNF-*α* (−308 G/A) was reported to be associated with T2DM among the Ethiopian population [58].

Moreover, all the variants of *TCF7L2* reported among Ghanaians [59], Cameroonians [60, 61], and Nigerians [62] were found to be significantly associated with T2DM. Among South Africa-Mixed Ancestry, *TCF7L2-rs7903146* showed marginal significance whereas *TCF7L2-rs12255372* was not significantly associated with T2DM [56].

### 3.6. Association between *TCF7L2-rs7903146* Risk Variant and T2DM among SSA Population

**Figure 5:**
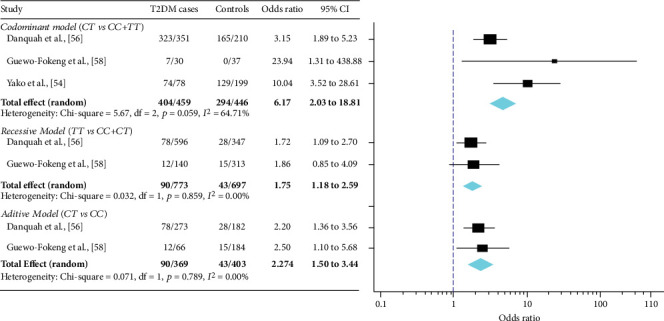
Forest plots for meta-analysis of the association between *TCF7L2-rs7903146* and the risk of T2DM among the SSA population.

The pooled estimate of the effect of the *TCF7L2-rs7903146* polymorphism on T2DM is shown in Figure 5. Across the three studies that assessed the effect of *TCF7L2-rs7903146* using the codominant genetic model, the pooled odds ratio was 6.17 (95% CI: 2.03-18.81; *I*^2^ = 64.71%, p − heterogeneity = 0.059). The effect of *TCF7L2-rs7903146* was 1.75 (95% CI: 1.18-2.59; *I*^2^ = 0.00%, p − heterogeneity = 0.859) using the recessive model across two studies; and 2.27 (95% CI: 1.50-3.44; *I*^2^ = 0.00%, p − heterogeneity = 0.789) additive model across two studies. These values suggest that the effect of *TCF7L2-rs7903146* on T2DM risk is moderately strong among the SSA population with moderate heterogeneity existing across studies.

## 4. Discussion

### 4.1. Pattern and Characteristics of T2DM Heritability in SSA

This review process provided a comprehensive and integrated perspective on heritability and genetic risk of T2DM in SSA. T2DM aggregation among the SSA population with PFH is 2.5 times the rate observed among the general population (28.2% vs. 11.2%). Likewise, the risk of T2DM increases to about 3.3-fold among the population with PFH compared with those without a family history of T2DM. Notwithstanding, significant heterogeneity existed among studies. Evidence from a variety of population, family, and twin-based studies [63, 64] has provided an estimated range for the heritability of T2DM to be 20%-80%, which is similar to that observed among SSA population. Another findings of Florez et al. [65] generally indicated that the risk of T2DM among the relative of individuals with T2DM is 3 times higher compared with individuals without a PFH. Thus, family history is not only an important risk factor for T2DM but also explains the importance of inheritance to the growing burden of T2DM among the population of SSA. It also represents a major role for inborn susceptibility to T2DM.

The features of T2DM heritability in SSA are of the preference of maternal to paternal aggregation and higher among first-degree relatives. These evidences have been confirmed by several studies across different population groups [26, 66–68]. However, some studies have reported no parental differences in disease transmission [63, 69, 70]. Earlier evidence suggested that considering the pattern of familial aggregation of T2DM, the possible explanations are true genetic maternal autosomal inheritance, which results from the passing on to offspring, maternally derived genetic variants in preference to paternal genes and/or maternal genes preferentially switched on during ontogeny [66, 71]. Based on current research evidence, it is possible that excess maternal influence may be explained by the intrauterine environment of the diabetic mother that may leave epigenetic signatures, which alter gene expressions responsible for insulin secretion, beta-cell and glucose uptake, and tolerance mechanisms [64]. Pettitt et al. [72] demonstrated that diabetic risk is high among the offspring of Pima Indian women who were diabetic during pregnancy than if the mothers develop diabetes later in life. In the present review, data were not available on the diabetic state of mothers at the time of pregnancy. Accordingly, a long-term prospective population study would be required to obtain these data with certainty, following up the offspring for the development of diabetes.

T2DM risk among the population with PFH acts through impaired insulin secretion as a result of beta-cell dysfunction rather than insulin action. Accordingly, genetic risk variants identified as ancestry-specific signals through fine mapping of causal variants (*AGMO-rs73284431*, *VT11A-rs17746147*, and *ZRANB3*), and the transferable genetic risk variants (*TCF7L2*), are reported to act through beta-cell dysfunction to confer the risk of T2DM [36, 37]. Moreover, linkage signals detected among the SSA population from the two maiden linkage studies [30, 31] were loci that contain genes involved in beta-cell function instead of insulin action. This establishes that inherited abnormalities of beta-cell function/mass are critical precursors of T2DM among the SSA population. Typical of T2DM phenotype in SSA, Kibirige et al. [73] in a narrative review indicated that hyperglycaemia in most cases are predominantly characterised by delayed acute first phase of insulin secretion and pancreatic beta-cell secretory dysfunction, rather than peripheral insulin resistance.

The inheritance basis of T2DM among the SSA population, like many other groups, is typical of multifactorial inheritance of several different genes and likely epi-alleles that affects beta-cell function [74]. In examining T2DM in SSA, Kibirige et al. [73] reported that chronic inflammation, early life malnutrition, and epigenetic modifications are potential contributors to the distinct differences in disease manifestation. It is also true that insulin resistance is a precursor for T2DM, and insulin activity may be subject to genetic variance at several loci. Thus, to understanding the details of the pattern and characteristics of T2DM heritability in SSA, detailed studies with the focus on the genetic characteristics of familial T2DM pedigrees, glucose transport and tolerance mechanisms, as well as insulin secretion mechanisms among T2DM probands (considering the effect of biological ageing and environmental stressors on the expression of insulin signalling proteins) will be useful.

### 4.2. Genetics Risk of T2DM in SSA

Existing studies in SSA have tested 27 polymorphisms in 16 genes that have been reported to be associated with T2DM in other population groups. The candidate gene study approach was used with trends mostly toward consistent effect for genes replicated in multiple studies and modest effect size of the variant with a positive signal. Regarding the effect of the European common variant (*TCF7L2*) and Asian common variant *KCNQ*) with the greatest effect, only *TCFL2* has been replicated among the SSA population and its role defined. Genome-wide association/linkage studies revealed linkage signals in chromosome regions harbouring these two genes; however, studies of selected SNPs found an almost consistent association of *TCF7L2* with T2DM whereas that of *KCNQ* was not replicated.

A meta-analysis of GWAS has consistently reported that some T2DM genetic risk signals are transferable across populations, whereas others are population-specific [75, 76].

We identified three studies that have explored the human genome using GWAS in the SSA population and uncovered evidence for three ancestry-specific signals (*AGMO*, *VT11A*, and *ZRANB3*), which are distinct for African population [35–37]. In addition, *TCF7L2* was identified to be transferable to populations within SSA. Heterogeneity studies of the effect of transferable genetic risk variants across the population have been consistent with the reporting of the effect estimate. Similarly, the effect estimate of *TCF7L2* among the SSA population was within the range reported among Asians and Europeans. Thus, there is the homogeneity of the effect of *TCF7L2* variants across the population, which is likely to project a vital contribution of differences in environmental factors to the T2DM prevalent rate.

Whether or not, and to what extent GWAS signals identified in other non-African populations would be transferable across SSA populations remains a question to be answered. Besides the evidence of the transferability of *TCF7L2* among the SSA population, others like *KCNJ11 (rs5219)* were among Mauritanians [55] but not Ghanaian [49] and Nigerian [54] populations. This has also been generally observed among the entire African population [77]. The inconsistencies are likely due to factors including differences in underlying genetic architecture, inadequate sample size, and genotyping methods. Further contributing to these inconsistencies, there exist a report of exaggerated effect estimates that is often seen in the earliest association studies compared with subsequent attempts to replicate the finding [78]. Overall, systematic approaches that take into consideration these limitations are needed to adequately answer the question of how well loci identified in non-Africa populations transfer across African populations. These systematic approaches have been applied in a few studies [35–37] and unravelled both global cosmopolitan and African specific alleles in T2DM aetiology.

### 4.3. Research and Clinical Perspectives on Heritability and Genetic Risk of T2DM in SSA

All the SNPs identified or replicated in SSA are within introns or intergenic regions. Accordingly, their functional significance remains to be resolved, and evidence suggests their involvement in the regulation of gene expression or splicing [79]. SNPs associated with common traits by far have been found to be enriched for expression quantitative trait loci (eQTLs) [80]. Hence, among the SSA population, additional insights on the genetic architecture of T2DM can be explored through transcriptomics, quantification, and expression profiling of genes in specific tissues using microarrays and high throughput RNA sequencing technologies (RNAseq).

One important question to consider among the SSA population is how well do the genetic and environmental factors interacts for T2DM expression and inheritance? Illustratively, evidence from nongenetic factors establishes a direct relationship between T2DM and insulin resistance, whereas heritability also presents a characteristic T2DM arising from beta-cell dysfunction. Therefore, genetic and nongenetic forms of T2DM can be characterized in SSA, where the disease can occur either in isolation or with affected children born to unaffected parents. If there is more to this, detailed analysis of epigenetic mechanisms in T2DM development in a larger sample size merits attention among SSA populations. Furthermore, the wide genetic diversity of the African population and differences in the distribution of nongenetic factors present researchers with opportunities to investigate gene-environment interaction (epigenetic) questions.

Matsha et al. [81] in a study among the Black South African population indicated that epigenetic changes are likely to be an early process that occurs before the onset of overt diabetes. Also, in the first Epigenome-wide Association Study in SSA, four novel differentially methylated probes (DMPs) at epigenome-wide level were identified, which provided insights into the epigenetic loci that underlie the burden of T2DM [82]. Demonstratively, palmitate exposure to human pancreatic islets induced global and specific DNA methylation alterations that resulted in coordinated changes in mRNA expression and decreased insulin secretion [83]. Therefore, better characterization of nongenetic risk factors and investigation of their synergistic effects with putative genetic risk variants will be needed for the proper tailoring of clinical and public health intervention strategies of T2DM in SSA.

## 5. Conclusion

This is the first meta-analysis to investigate the heritability and genetic risk of T2DM among SSA population. The current evidence on the heritability and genetic markers of T2DM in SSA populations is limited and largely insufficient to reliably inform the genetic architecture of T2DM across SSA regions and to inform clinical management of T2DM. Thus, studies to provide ample data in the areas of heritability, family history, and genetic risk of T2DM are warranted.

## Figures and Tables

**Table 1:** shows the prevalence of T2DM among participants with a positive family history of T2DM.

Study	Sample size	PFH population		NFH population	
		Prevalence (%)	95% CI	Prevalence (%)	95% CI
Bantie et al. [38]	607	19.2	11.2-29.7	8.9	6.6-11.6
Zenebe et al. [39]	264	35.1	20.2-52.5	15.9	11.4-21.3
Bello-Ovosi et al. [40]	172	35.5	19.2-54.6	22.0	15.5-29.7
Wondemagegn et al. [41]	714	23.1	17.0-30.1	8.1	5.9-10.7
Tesfaye et al. [42]	851	19.6	10.2-32.4	4.2	2.9-5.8
Vuvor et al. [43]	597	14.5	7.2-25.0	2.5	1.3-4.2
Millogo et al. [44]	4415	5.4	2.7-9.4	5.5	4.8-6.2
Abebe et al. [45]	2136	17.9	10.2-28.3	3.1	2.4-3.9
Mayega et al. [46]	1497	20.9	15.2-27.5	15.1	13.2-17.2
Nyenwe et al. [47]	492	26.3	13.4-43.1	5.3	3.4-7.8
Frank et al. [48]	1221	65.0	60.54-69.19	30.7	27.37-34.18
Danquah et al. [49]	1466	66.8	62.86-70.65	32.2	29.16-35.44
Total (random)	14,432	28.2	14.52-44.27	11.2	6.39-17.06
*I* ^2^ (inconsistency) = 98.11%, *p* < 0.0001					

PFH: positive family history; NFH: negative family history.

## Supplementary Materials

Supplementary Materials Supplementary Appendix 1: methodological details and characteristics of included studies (Supplementary Materials).
