# Supplementary material for: Heritability and Genetics of Type 2 Diabetes Mellitus in Sub-Saharan Africa: A Systematic Review and Meta-Analysis
Source: J Diabetes Res. 2020 Jun 30;2020:3198671. doi: 10.1155/2020/3198671 (PMC7352126; doi:10.1155/2020/3198671)
Supplement: Supplementary Materials — Supplementary Appendix 1: methodological details and characteristics of included studies (Supplementary Materials). [file 3198671.f1.pdf]

Heritability and Genetics of Type 2 Diabetes Mellitus in Sub-Saharan Africa: A systematic Review and Meta-analysis

Supplementary Appendix

Table of Contents

Table S1: PubMed general Search Strategy 1

SCOPUS Search Strategy *on* epidemiology/aetiology of T2DM in SSA 1

Table S2: PubMed Search Strategy on Genetic risk of T2DM in SSA 2

SCOPUS Search Strategy on Genetic risk of T2DM in SSA 2

Table S3: Web of Science (Science Citation Index Expanded) Search Strategy 3

Figure S1: Time trend in genetic, FH and heritability studies of T2DM in SSA........ 3

Eligibility of Articles and Articles Selection 3

Protocol for Abstract/Title Screening 5

Protocol for Full Text Review 5

Data extraction Protocol........ 6

Fig S2: Conceptual framework of the review process. 7

Table S4: Polymorphisms investigated in genetic studies of T2DM in SSA until December 2019 8

Table S5 Characteristics of studies on family history and Risk of T2DM in SSA until December 2019........ 12

References 15

Table S1: PubMed general Search Strategy

Search	Query	Number of hits
#1	"Diabetes Mellitus/epidemiology"[Mesh] OR "Diabetes Mellitus, Type 2/etiology"[Mesh] OR "non-insulin dependent diabetes mellitus/epidemiology"[tiab] OR "non-insulin dependent diabetes mellitus/aetiology"[tiab])	68335
#2	(("Africa South of the Sahara"[Mesh] OR "ivory coast"[tiab] OR Cameroon[tiab] OR Central African Republic[tiab] OR Chad[tiab] OR Congo[tiab] OR Democratic Republic of the Congo[tiab] OR Equatorial Guinea[tiab] OR Gabon[tiab] OR Burundi[tiab] OR Eritrea[tiab] OR Ethiopia[tiab] OR Kenya[tiab] OR Rwanda[tiab] OR Sudan[tiab] OR Tanzania[tiab] OR Uganda[tiab] OR Angola[tiab] OR Botswana[tiab] OR Lesotho[tiab] OR Malawi[tiab] OR Mozambique[tiab] OR Namibia[tiab] OR South Africa[tiab] OR Swaziland[tiab] OR Zambia[tiab] OR Zimbabwe[tiab] OR Benin[tiab] OR Burkina Faso[tiab] OR Cape Verde[tiab] OR Cote d'Ivoire[tiab] OR Gambia[tiab] OR Ghana[tiab] OR Guinea[tiab] OR Guinea-Bissau[tiab] OR Liberia[tiab] OR Mali[tiab] OR Mauritania[tiab] OR Niger[tiab] OR Nigeria[tiab] OR Senegal[tiab] OR Sierra Leone[tiab] OR Togo[tiab] OR (subsaharan[tiab] AND africa[tiab]))	373327
#3	("2000/01/01"[PDat]: "2019/12/31"[PDat])	16912239
#4	#1 AND #2 AND #3	621

SCOPUS Search Strategy *on* epidemiology/aetiology of T2DM in SSA

Query String (2581 items)

(Epidemiology* OR prevalence* AND "diabetes mellitus" OR "type 2 diabetes mellitus" OR "non-insulin dependent diabetes mellitus")
AND
(LIMIT-TO (AFFILCOUNTRY , "South Africa") OR LIMIT-TO (AFFILCOUNTRY , "Nigeria") OR LIMIT-TO (AFFILCOUNTRY , "Ethiopia") OR LIMIT-TO (AFFILCOUNTRY , "Ghana") OR LIMIT-TO (AFFILCOUNTRY , "Cameroon") OR LIMIT-TO (AFFILCOUNTRY , "Kenya") OR LIMIT-TO (AFFILCOUNTRY , "Tanzania") OR LIMIT-TO (AFFILCOUNTRY , "Uganda") OR LIMIT-TO (AFFILCOUNTRY , "Mauritius") OR LIMIT-TO (AFFILCOUNTRY , "Congo") OR LIMIT-TO (AFFILCOUNTRY , "Senegal") OR LIMIT-TO (AFFILCOUNTRY , "Malawi") OR LIMIT-TO (AFFILCOUNTRY , "Cote d'Ivoire") OR LIMIT-TO (AFFILCOUNTRY , "Zimbabwe") OR LIMIT-TO (AFFILCOUNTRY , "Benin") OR LIMIT-TO (AFFILCOUNTRY , "Gambia") OR LIMIT-TO (AFFILCOUNTRY , "Mozambique") OR LIMIT-TO (AFFILCOUNTRY , "Zambia") OR LIMIT-TO (AFFILCOUNTRY , "Botswana") OR LIMIT-TO (AFFILCOUNTRY , "Burkina Faso") OR LIMIT-TO (AFFILCOUNTRY , "Rwanda") OR LIMIT-TO (AFFILCOUNTRY , "Guinea") OR LIMIT-TO (AFFILCOUNTRY , "Democratic Republic Congo") OR LIMIT-TO (AFFILCOUNTRY , "Mali") OR LIMIT-TO (AFFILCOUNTRY , "Togo")) AND (LIMIT-TO (DOCTYPE, "ar"))

Table S2: PubMed Search Strategy on Genetic risk of T2DM in SSA

Search	Query	Number of hits
#1	(genetics*[Mesh]] OR "genetic marker[tiab]" OR "genetic polymorph*[tiab]" OR "single nucleotide polymorphism [tiab]" OR “candidate gene polymorphism[tiab]” OR gene* [tiab] OR allele [tiab])	4290283
#2	("Diabetes mellitus"[Mesh] OR “Type 2 diabetes mellitus[tiab]”)	127095
#3	(("Africa South of the Sahara"[Mesh] OR "ivory coast"[tiab] OR Cameroon[tiab] OR Central African Republic[tiab] OR Chad[tiab] OR Congo[tiab] OR Democratic Republic of the Congo[tiab] OR Equatorial Guinea[tiab] OR Gabon[tiab] OR Burundi[tiab] OR Eritrea[tiab] OR Ethiopia[tiab] OR Kenya[tiab] OR Rwanda[tiab] OR Sudan[tiab] OR Tanzania[tiab] OR Uganda[tiab] OR Angola[tiab] OR Botswana[tiab] OR Lesotho[tiab] OR Malawi[tiab] OR Mozambique[tiab] OR Namibia[tiab] OR South Africa[tiab] OR Swaziland[tiab] OR Zambia[tiab] OR Zimbabwe[tiab] OR Benin[tiab] OR Burkina Faso[tiab] OR Cape Verde[tiab] OR Cote d'Ivoire[tiab] OR Gambia[tiab] OR Ghana[tiab] OR Guinea[tiab] OR Guinea-Bissau[tiab] OR Liberia[tiab] OR Mali[tiab] OR Mauritania[tiab] OR Niger[tiab] OR Nigeria[tiab] OR Senegal[tiab] OR Sierra Leone[tiab] OR Togo[tiab] OR (subsaharan[tiab] AND africa[tiab]))	373327
#4	(“2000/01/01"[PDat]: "2019/12/31"[PDat])	16912239
#5	#1 AND #2 AND #3 AND #4	102

SCOPUS Search Strategy on Genetic risk of T2DM in SSA

Query string (213 records)
"genetic risk" OR "single nucleotide polymorphisms" OR "candidate gene polymorphisms" OR AND "diabetes mellitus" OR "type 2 diabetes mellitus" OR "non-insulin dependent diabetes mellitus"
AND
(LIMIT-TO (AFFILCOUNTRY , "South Africa") OR LIMIT-TO (AFFILCOUNTRY , "Nigeria") OR LIMIT-TO (AFFILCOUNTRY , "Ghana") OR LIMIT-TO (AFFILCOUNTRY , "Cameroon") OR LIMIT-TO (AFFILCOUNTRY , "Uganda") OR LIMIT-TO (AFFILCOUNTRY , "Ethiopia") OR LIMIT-TO (AFFILCOUNTRY , "Tanzania") OR LIMIT-TO (AFFILCOUNTRY , "Mauritius") OR LIMIT-TO (AFFILCOUNTRY , "Gambia") OR LIMIT-TO (AFFILCOUNTRY , "Kenya") OR LIMIT-TO (AFFILCOUNTRY , "Mozambique") OR LIMIT-TO (AFFILCOUNTRY , "Guinea") OR LIMIT-TO (AFFILCOUNTRY , "Botswana") OR LIMIT-TO (AFFILCOUNTRY , "Malawi") OR LIMIT-TO (AFFILCOUNTRY , "Mali") OR LIMIT-TO (AFFILCOUNTRY , "Senegal") OR LIMIT-TO (AFFILCOUNTRY , "Zimbabwe") OR LIMIT-TO (AFFILCOUNTRY , "Benin") OR LIMIT-TO (AFFILCOUNTRY , "Congo") OR LIMIT-TO (AFFILCOUNTRY , "Cote d'Ivoire") OR LIMIT-TO (AFFILCOUNTRY , "Gabon") OR LIMIT-TO (AFFILCOUNTRY , "Guinea-Bissau") OR LIMIT-TO (AFFILCOUNTRY , "Rwanda")

Table S3: Web of Science (Science Citation Index Expanded) Search Strategy

Search	Query	Number of hits
#1	**TS**=(genetics OR genetic marker OR genetic polymorph* OR single nucleotide polymorphism OR candidate gene polymorphism OR gene* OR allele)	6,064,342
#2	**TS**=("Diabetes mellitus near/3 Type 2)	55,527
#3	**CU**=(Africa South of the Sahara OR ivory coast OR Cameroon OR Central African Republic OR Chad OR Congo OR Democratic Republic of the Congo OR Equatorial Guinea OR Gabon OR Burundi OR Eritrea OR Ethiopia OR Kenya OR Rwanda OR Sudan OR Tanzania OR Uganda OR Angola OR Botswana OR Lesotho OR Malawi OR Mozambique OR Namibia OR South Africa OR Swaziland OR Zambia OR Zimbabwe OR Benin OR Burkina Faso OR Cape Verde OR Cote d'Ivoire OR Gambia OR Ghana OR Guinea OR Guinea-Bissau OR Liberia OR Mali OR Mauritania OR Niger OR Nigeria OR Senegal OR Sierra Leone OR Togo OR sub Saharan OR Africa)	539,573
#4	Timespan= 2000-2019	
#5	#1 AND #2 AND #3 AND #4	114

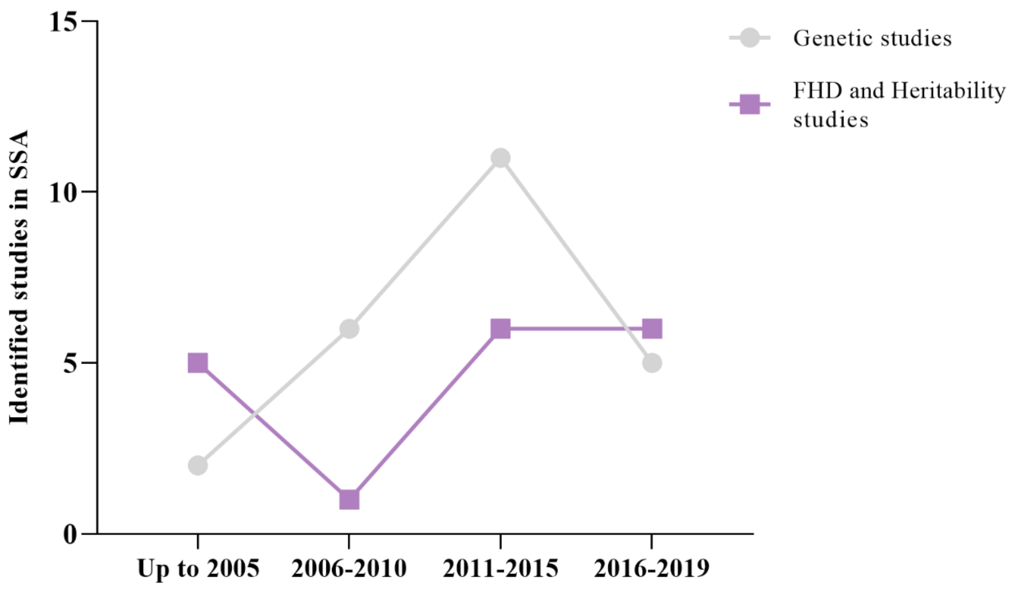

Figure S1: Time trend in genetic, FH and heritability studies of T2DM in SSA.

Eligibility of Articles and Articles Selection

Titles and abstracts of articles were reviewed independently by 2 reviewers, strictly using the inclusion criteria stated below. Selected articles underwent a second review-- a

full independent reading of each article by each reviewer. Additionally, the reference list of selected articles were reviewed for additional articles meeting inclusion criteria. Any disagreements regarding the inclusion/exclusion of an article was settled by consensus. Alternatively, a 3rd independent reviewer (MAA) was invited and a Cohen kappa coefficient was reported.

Inclusion criteria

1. **Design:** Non-randomized studies including cross-sectional studies (community-based, population-based, hospital based and comparative), case-control, and prospective cohort studies that reported a heritability, family history and genetic risk of T2DM.

   Rational: Majority of the total body of evidence on family history and risk of T2DM came from non-randomized control studies and they represented the major body of evidence on demographic factors associated with T2DM in SSA. Besides, case-control studies represented the ideal design for genetic association studies. Therefore, for comprehensive review, we chose to include all study designs listed above.

2. **Outcomes:** Definition, diagnosis and classification of T2DM was based on the 1997 American Diabetes Association and 1999 World Health Organization criteria for hyperglycemia in the diagnosis and prediction of diabetes [1].

   Special consideration: Candidate gene studies should have value for Hardy Weinberg equilibrium (HWE), and effect estimate adjusted for covariates. For family history and T2DM risk, studies should have effect estimate for PFH adjusted for covariate.

3. **Definitions:** Terms such as "type-2 diabetes mellitus", "Type 2 Diabetes", "Diabetes Mellitus, Slow Onset", "Noninsulin-Dependent Diabetes Mellitus", "Maturity Onset Diabetes Mellitus", "Diabetes Mellitus, Stable", "Diabetes Mellitus, Type II", "Adult-Onset Diabetes Mellitus", "Diabetes Mellitus, Noninsulin Dependent", "NIDDM", "Diabetes, Maturity-Onset", "Diabetes, Type 2", "Slow-Onset Diabetes Mellitus", "Diabetes Mellitus, Non-Insulin-Dependent", "Diabetes Mellitus, Ketosis-Resistant", "Ketosis-Resistant Diabetes Mellitus", and "Maturity-Onset Diabetes Mellitus" were included under the umbrella of diabetes mellitus, type 2.

4. **Participants/sample:** All populations and sub-populations should be of either sex but not limited to the general population, outpatient clinics, T2DM, hypertensives, etc.

    Special consideration: Candidate gene association studies should include T2DM cases and aged and sex matched non-diabetic control group. Familial studies should include offspring (case subjects) of families with at least one T2DM parent and offspring (control subjects) from families with no T2DM parents.

Protocol for Abstract/Title Screening

Based on the inclusion criteria, there were 6 yes/no questions for the abstract/title screening process. If all questions were answered yes (or maybe), the article was included for full text review. Questions that could not be satisfactorily answered from the title or abstract alone for a particular study was included for full text review.

1. Did the article take place and study persons in sub-Saharan Africa?
2. Is the study design consistent with our defined inclusion criteria for study design?
3. Does the classification and definition of T2DM in some capacity as defined by the outcomes section of the inclusion criteria?
4. Did demographic data include family history of T2DM?
5. Does the study involve common genetic variants or risk of T2DM among probands?

Protocol for Full Text Review

Based on the inclusion criteria, there were a series of yes/no questions for the full article text review under specific headings. If questions #1 (general) and #2 (under specific headings) were answered yes, then the article was included for quantitative data extraction.

General

1. Does the population and study design in fact meet all of our inclusion criteria as answered in the abstract/title review?

Family history and risk of T2DM

2. Did the article report PFH as a risk factor for T2DM with available data?

Candidate gene study

2. Does the study provides the number of genotypes in case-controls groups for calculating Odds Ratios (ORs)?

Special considerations: studies on heritability and GWAS were included in a narrative review.

Data extraction Protocol

After identifying articles for inclusion, two authors (EA and CO) independently reviewed each article for data extraction into a standard, pre-formulated form: ***Family history of T2DM:*** data was extracted under the following headings: name of first author, publication year, sub-Saharan Africa region, country, and study design, participants, sampling technique, and sample size, criterial for T2DM definition, percentage of participants with positive family history (PFH), T2DM prevalence in PFH and T2DM prevalence in negative family history.

Gene-association study: data was extracted under the headings- first author and publication year; population; polymorphic variant; total sample size; the number of nucleotide polymorphisms genotypes both in cases and controls, genotyping method; minor allele frequency. For the polymorphisms investigated in multiple studies, we derived the pooled estimates of their association with T2DM risk across studies using a random and mixed effects model meta-analysis, implemented using MedCalc Software for Windows, version 18.91 (https://www.medcalc.org/). Fig S2 gives a summary of the review process.

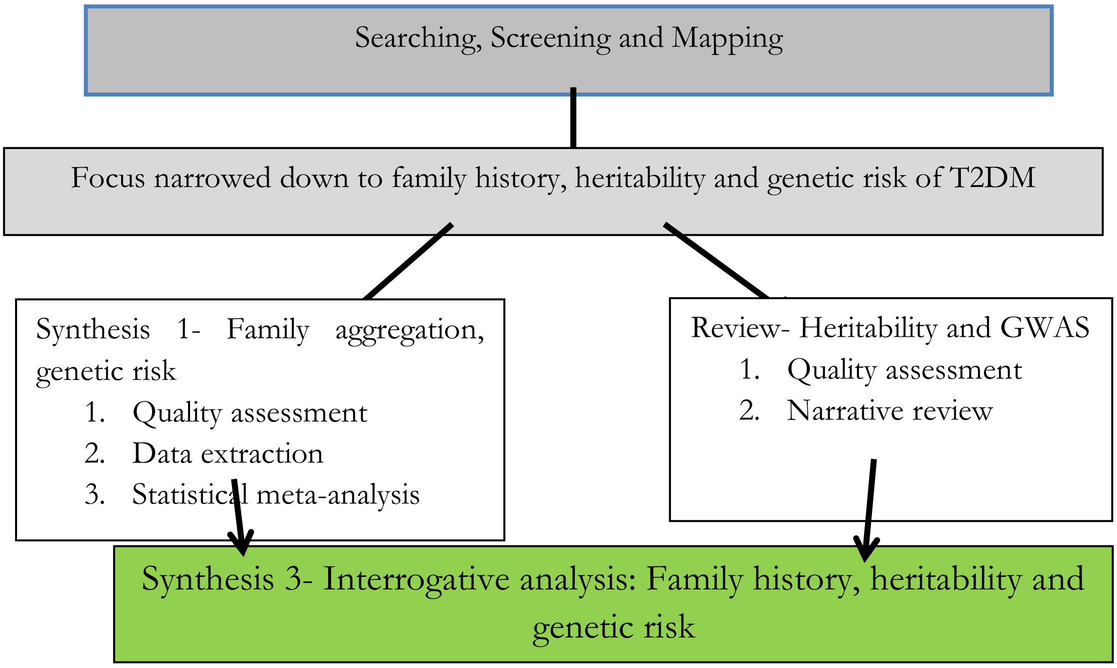

Fig S2: Conceptual framework of the review process.

Table S4: Polymorphisms investigated in genetic studies of T2DM in SSA until December 2019

Study	Population	Sample size	Number of case/controls	Genotyping method	Gene	Polymorphism	Risk allele	MAF-Cases	MAF-Controls	HWE	Effect estimate	Adjustment
Chen *et al.,* [2]	Nigerian (Igbo and Yuroba)	294	197/97	Pyrosequencing	*CAPN10*	*rs3792267*	G	0.81	0.86	No deviation	na	na
						rs2975762	A	0.74	0.77		na	
						rs5030952	C	0.46	0.44		na	
	Ghanaian (Akan-Ashante and Ga	201	150/51	Pyrosequencing	*CAPN10*	*rs3792267*	G	0.9	0.88	No deviation	na	
						rs2975762	A	0.79	0.71		na	
						rs5030952	C	0.46	0.46		na	
Danquah *et al.,* [3]	Ghanaian	1049	674/375	PCR-RFLP	*TCF7L2*	*rs7903146*	T	0.36	0.3	No deviation	OR (additive model): 1.39 (1.07–1.81) p=0.014	Age, sex, BMI, HTN
		1052	675/377		*KCNJ11*	*rs5219*	G	0.0007	0		Not significant	
		1021	656/365		*PPAR-γ*	*rs1801282*	G	0.0008	0		Not significant	
		1048	674/375		*CAPN10*	*rs3842570*	3 repeats	0.23	0.23		OR (additive model)= 1.12 (0.84–1.49), p=0.45	
			673/375			*rs3792267*	A	0.11	0.13		OR (additive model) = 0.70 (0.47–1.03) p=0.07	
			674/374			*rs5030952*	T	0.54	0.55		OR (additive model)= 0.98 (0.76–1.25) p=0.84	
Olckers *et al.,* [4]	Black sourth Africans	453	227/226	Real-Time PCR	*ACDC*	*SNP C-11377G*	G	0.137	0.154	No deviation	OR (CG)= 1.09 (0.72-1.65), p<0.19	None
						SNP G-11391A	A	0.004	0.026		OR =0.15 (0.03-0.71), p=0.0065	

Abdelhamid *et al.,* [5]	Mauritanian	270	135/135	TaqMan allelic discrimination	*KCNJ11*	*rs5219*	K (G)	0.218	0.156	No deviation	OR (general model): 2.08 (1.09–3.97) p=0.026; OR (additive model, AG): 2.27 (0.98-5.26) p=0.057; OR (additive model, GG): 3.35 (0.67-16.6) p=0.139; OR (dominant model): 2.49 (1.12-5.55) p=0.026; OR (recessive model): 2.73 (0.54-13.75) p=0.224	Age and Sex
Guewo-Fokeng *et al.,* [6]	Cameroonian	74	37/37	PCR-RFLP	*TCF7L2*	*rs7903146*	C	0.095	0	0.670	OR (additive model)= 18.44 (1.01-335.97), p=0.011	None
Yako *et al.,* [7]	South African mixed-ancestry	480	152/328	Taqman genotyping	*ENPPI*	*rs997509*	T	0.118	0.119	>0.999	OR (recessive model)= 4.60 (1.07-19.86), p= 0.040)	Age, sex, BMI, Insulin resistance
						rs1044498	A	0.49	0.486	0.269	Not significant	
					FTO	*rs9941349*	T	0.303	0.25	0.463	OR (additive model)= 1.43 (1.00-2.04), p= 0.052	
						rs3751812	T	0.214	0.182	0.854	Not significant	
					TCf7L2	*rs12255372*	T	0.23	0.186	0.200	Not significant	
						rs7903146		0.322	0.243	0.231	OR (additive model)= 1.43 (1.00-2.04), p= 0.053	
Engwa *et al.,* [8]	Nigerian	148	73/75	PCR-RFLP	*KCNJ11*	*rs5219*	K (A)	0.932	0.92	0.79	OR (AA): 1.183 (0.35-4.06), p=0.790	None
Mato *et al.,* [9]	Cameroonian	180	60/120	PCR-RFLP	*PPRG-γ*	*rs1801282*	G	0	0	No deviation	na	na

Engwa *et al.,* [10]	Nigerian	480	73/75	PCR-RFLP	*ABCC8*	*C49620T*	T	0.671	0.553	0.249	OR (codominant model)= 1.04 (1.01-1.07), p=0.009; OR (Dominant model)= 2.13 (1.00-4.52), p=0.05 OR (Recessive model)= 2.39 (1.16-4.91), p=0.018 OR (General mode, TTl)= 2.58 (1.15-5.77), p=0.021	Age
Quaye *et al.,* [11]	Ghanaian	216	129/87	LDR-PCR	*Haptoglobin*	*Hp2-2*	Hp2-2	0.348	0.155	No deviation	OR (Hp 2-2,)= 6.1 (1.8-21.2), P = 0.001	None
Katchunga *et al.,* [12]	Congolese (Bukawu)	265	179/86	PCR-RFLP	*Ferrpprotein*	*Q248H*	T	0.14	0.081	No deviation	OR = 1.70 (0.52–5.58), p= 0.370	Age, MetS
Lopez-Sall *et al.,* [13]	Senegalese	271	143/128	TaqMan allelic discrimination	*PPARα*	*rs1800206*	V (not reported)	na	na	na	na	na
Vergotine *et al.,* [14]	South African mixed-ancestry	787	212/575	Real-Time PCR	*PPRG-γ*	*rs1801282*	G	0.068	0.048	0.719	OR (additive model C)= 1.40 (0.85-2.28), p=0.176	Age and sex
					IRS-1	*Gly972Arg*	A	0.031	0.044	>0.999	OR (additive model)0.67 (0.34-1.24) p=0.228	
Bonilla *et al.,* [15]	West Africans	538	381/157	Pyrosequencing	*AGRP*	*AGRP38*	T	na	na	No deviation	OR: 0.86 (0.56–1.32) p=0.49	Age and BMI
Helgason *et al.,* [16]	West Africans	1069	621/448	Sequencing	*TCF7L2*	*DG10S478*	X	na	na	No deviation	RR 1.20 (0.91–1.59) p= 0.19	Relatedness and ancestry
						rs12255372	T	na	na		RR 1.31 (1.01–1.69) p= 0.044	
						rs7903146	T	na	na		RR 1.45 (1.19–1.77) p= 0.00021	

Vergotine *et al.,* [17]	South African mixed-ancestry	856	619/237	taqmam genotyping and sequecing	*IRS-1*	*Gly972Arg*	A	na	na	No deviation	Not significant	na
Nanfa *et al.,* [18]	Cameroonian	115	57/58	PCR-RFLP	*TCF7L2*	*rs12255372*	T	0.440	0.167	No deviation	OR (TT)= 4.33 (1.57 – 11.92), p=0.005	Age
**Chikowore *et al.,* [19]**	**Black South Africans**	**356**	**178/178**	**BeadXpress platform**	**PSMD6**	**rs831571**	**T**	**na**	**na**	**No deviation**	OR (Additive model)= 0.56 (0.34-0.92), p= 0.03*	**na**
					**FTO**	**rs8050136**	**A**	**na**	**na**		OR (Dominant model)= 0.59 (0.36-0.97), p= 0.04*	
					**SLC44A3, F3**	**rs7542900**	**T**	**na**	**na**		OR (recessive model)= 0.51 (0.28-0.91), p= 0.002*	
					**C2CD4B**	**rs1436955**	**C**	**na**	**na**		OR (Additive Model)= 1.46 (1.04-2.04), p= 0.03*	
**Ayelign *et al.,* [20]**	**Ethiopian**	**150**	**75/75**	**ARMS**	**TNF-α gene**	**−308 G/A**	**A**	**0.480**	**0.746**	**No deviation**	OR (Dominant model)= 2.67 (1.31-5.46) p=0.005; OR (codominant model) = 0.51(0.248–1.043) p=0.066; OR (Recessive model)= 0.457 (0.142–1.413),p=0.208; OR (Allelic model)= 0.474 (0.279–0.802), p=0.002	**na**

***- association was not significant after correcting for multiple testing, na- not available. Highlighted SNPs were significantly associated with T2DM among SSA population**

Table S5 Characteristics of studies on family history and Risk of T2DM in SSA until December 2019

Study	Year	Region	Country	Design	Participants	Sampling technique	Sample size	PFH (%)	Criteria
Danquah *et al.,* [21]	2012	West Africa	Ghana	Hospital based case-control study	T2D, HTN, outpatients, community and hospital staff	Multi-stage random	1466	44.9%	WHO, 1999
Frank *et al.,* [22]	2012	West Africa	Ghana	Hospital based case-control study	T2D, HTN, outpatients, community and hospital staff	Multi-stage random	1221	37.0%	WHO, 1999
Vuvor *et al.,* [23]	2011	West Africa	Ghana	Population-based study cross-sectional study	Urban and peri-urban communities	Multi-stage random	597	11.5%	WHO, 1999
Bantie *et al.,* [24]	2019	East Africa	Ethiopia	Community-based cross-sectional study	Adults aged 18 years and above	Multi-stage random	607	12.9%	Not specified
Zenebe *et al.,* [25]	2019	East Africa	Ethiopia	Community-based cross-sectional study	Adult aged 40 years and above	Random sampling	264	14.0%	WHO, 1999

Millogo *et al.,* [26]	2018	West Africa	Burkina Faso	Population-based cross-sectional study	Adults population	Multi-stage random	4417	4.8%	WHO, 1999
Bello-Ovosi *et al.,* [27]	2018	West Africa	Nigeria	Community-based cross-sectional study	Adults aged 18 years	Random sampling	172	18.2%	ADA, 2016
Wondemagegn *et al.,* [28]	2017	East Africa	Ethiopia	Community-based cross-sectional study	Adult aged 25 and above	Multi-stage random	757	23.7%	WHO, 1999
Tesfaye *et al.,* [29]	2016	East Africa	Ethiopia	Community-based Cross-sectional study	Federal police commission residing in Addis Ababa	Multi-stage random	936	6.0%	WHO, 1999
Abebe *et al.,* [30]	2014	East Africa	Ethiopia	Population-based cross-sectional study	Adults aged 35 years and above	Multi-stage random	2141	3.6%	WHO, 1999
Mayega *et al.,* [31]	2013	Central Africa	Uganda	Population-based cross-sectional study	Adults aged 35-60 years	Multi-stage random	1,497	12.2%	WHO, 2006; ADA, 2010
Chege [32]	2010	East Africa	Kenya	cross-section comparative study	Outpatient	Simple Random	90		Not specified
Nyenwe *et al.,* [33]	2003	West Africa	Nigeria	Population-based cross-sectional study	Adults aged 40 years and above	Multi-stage random	502	7.6%	WHO, 1999

References

1. Wareham NJ, O'Rahilly S. The changing classification and diagnosis of diabetes: new classification is based on pathogenesis, not insulin dependence. British Medical Journal Publishing Group; 1998.
2. Chen Y, Kittles R, Zhou J, Chen G, Adeyemo A, Panguluri RK, et al. Calpain-10 gene polymorphisms and type 2 diabetes in West Africans: the Africa America Diabetes Mellitus (AADM) Study. Annals of epidemiology. 2005;15(2):153-9.
3. Danquah I, Othmer T, Frank LK, Bedu-Addo G, Schulze MB, Mockenhaupt FP. The TCF7L2 rs7903146 (T) allele is associated with type 2 diabetes in urban Ghana: a hospital-based case–control study. BMC medical genetics. 2013;14(1):96.
4. Olckers A, Towers GW, Van der Merwe A, Schwarz PE, Rheeder P, Schutte AE. Protective effect against type 2 diabetes mellitus identified within the ACDC gene in a black South African diabetic cohort. Metabolism. 2007;56(5):587-92.
5. Abdelhamid I, Lasram K, Meiloud G, Halim NB, Kefi R, Samb A, et al. E23K variant in KCNJ11 gene is associated with susceptibility to type 2 diabetes in the Mauritanian population. Primary Care Diabetes. 2014;8(2):171-5.
6. Guewo-Fokeng M, Sobngwi E, Atogho-Tiedeu B, Donfack OS, Noubiap JJN, Ngwa EN, et al. Contribution of the TCF7L2 rs7903146 (C/T) gene polymorphism to the susceptibility to type 2 diabetes mellitus in Cameroon. Journal of Diabetes & Metabolic Disorders. 2015;14(1):26.
7. Yako YY, Madubedube JH, Kengne AP, Erasmus RT, Pillay TS, Matsha TE. Contribution of ENPP1, TCF7L2, and FTO polymorphisms to type 2 diabetes in mixed ancestry ethnic population of South Africa. African health sciences. 2015;15(4):1149-60.
8. Engwa GA, Nwalo FN, Obi CE, Onyia C, Ojo OO, Mbacham W, et al. Predominance of the a allele but no association of the KCNJ11 rs5219 E23K polymorphism with type 2 diabetes in a Nigerian population. 2018.
9. Mato EPM, Pokam-Fosso PE, Atogho-Tiedeu B, Noubiap JJN, Evehe M-S, Djokam-Dadjeu R, et al. The Pro12Ala polymorphism in the PPAR-γ2 gene is not associated to obesity and type 2 diabetes mellitus in a Cameroonian population. BMC obesity. 2016;3(1):26.
10. Engwa GA, Nwalo FN, Chikezie CC, Onyia CO, Ojo OO, Mbacham WF, et al. Possible association between ABCC8 C49620T polymorphism and type 2 diabetes in a Nigerian population. BMC medical genetics. 2018;19(1):78.
11. Quaye IK, Ababio G, Amoah AG. Haptoglobin 2-2 phenotype is a risk factor for type 2 diabetes in Ghana. Journal of atherosclerosis and thrombosis. 2006;13(2):90-4.
12. Katchunga PB, Baguma M, Jean-René M, Philippé J, Hermans MP, Delanghe J. Ferroportin Q248H mutation, hyperferritinemia and atypical type 2 diabetes mellitus in South Kivu. Diabetes & Metabolic Syndrome: Clinical Research & Reviews. 2013;7(2):112-5.
13. Lopez-Sall P, Bard J, Nazih H, Cissé A, Bobin-Dubigeon C, Sall N, editors. PPAR alpha-L162V, frequency and relationship with type 2 diabetes among black Senegalese people. Annales de biologie clinique; 2008.
14. Vergotine Z, Yako YY, Kengne AP, Erasmus RT, Matsha TE. Proliferator-activated receptor gamma Pro12Ala interacts with the insulin receptor substrate 1 Gly972Arg and increase the risk of insulin resistance and diabetes in the mixed ancestry population from South Africa. BMC genetics. 2014;15(1):10.

15. Bonilla C, Panguluri R, Taliaferro-Smith L, Argyropoulos G, Chen G, Adeyemo A, et al. Agouti-related protein promoter variant associated with leanness and decreased risk for diabetes in West Africans. International journal of obesity. 2006;30(4):715.
16. Helgason A, Pálsson S, Thorleifsson G, Grant SF, Emilsson V, Gunnarsdottir S, et al. Refining the impact of TCF7L2 gene variants on type 2 diabetes and adaptive evolution. Nature genetics. 2007;39(2):218.
17. Vergotine Z, Kengne AP, Erasmus RT, Matsha TE. No evidence for association of insulin receptor substrate-1 Gly972Arg variant with type 2 diabetes mellitus in a mixed-ancestry population of South Africa. SAMJ: South African Medical Journal. 2014;104(6):420-3.
18. Nanfa D, Sobngwi E, Atogho-Tiedeu B, Noubiap JJN, Donfack OS, Mofo EPM, et al. Association between the TCF7L2 rs12255372 (G/T) gene polymorphism and type 2 diabetes mellitus in a Cameroonian population: a pilot study. Clinical and translational medicine. 2015;4(1):17.
19. Chikowore T, Conradie KR, Towers GW, van Zyl T. Common variants associated with type 2 diabetes in a Black South African population of Setswana descent: African populations diverge. Omics: a journal of integrative biology. 2015;19(10):617-26.
20. Ayelign B, Genetu M, Wondmagegn T, Adane G, Negash M, Berhane N. TNF-alpha (-308) Gene Polymorphism and Type 2 Diabetes Mellitus in Ethiopian Diabetes Patients. Diabetes Metabolic Syndrome and Obesity-Targets and Therapy. 2019;12:2453-9. doi: 10.2147/dmso.S229987. PubMed PMID: WOS:000504029700001.
21. Danquah I, Bedu-Addo G, Terpe K-J, Micah F, Amoako YA, Awuku YA, et al. Diabetes mellitus type 2 in urban Ghana: characteristics and associated factors. BMC public health. 2012;12(1):210.
22. Frank LK, Heraclides A, Danquah I, Bedu-Addo G, Mockenhaupt FP, Schulze MB. Measures of general and central obesity and risk of type 2 diabetes in a Ghanaian population. Tropical Medicine & International Health. 2013;18(2):141-51.
23. Vuvor F, Steiner-Asiedu M, Armar-Klemesu M, Armah S. Population-based study of diabetic mellitus prevalence and its associated factors in adult Ghanaians in the greater Accra region. International Journal of Diabetes in Developing Countries. 2011;31(3):149.
24. Bantie GM, Wondaye AA, Arike EB, Melaku MT, Ejigu ST, Lule A, et al. Prevalence of undiagnosed diabetes mellitus and associated factors among adult residents of Bahir Dar city, northwest Ethiopia: a community-based cross-sectional study. BMJ open. 2019;9(10).
25. Zenebe T, Merga H, Habte E. A community-based cross-sectional study of magnitude of dysglycemia and associated factors in Southwest Ethiopia. International Journal of Diabetes in Developing Countries. 2019:1-7.
26. Millogo T, Bicaba BW, Soubeiga JK, Dabiré E, Médah I, Kouanda S. Diabetes and abnormal glucose regulation in the adult population of Burkina Faso: prevalence and predictors. BMC public health. 2018;18(1):350.
27. Bello-Ovosi BO, Asuke S, Abdulrahman SO, Ibrahim MS, Ovosi JO, Ogunsina MA, et al. Prevalence and correlates of hypertension and diabetes mellitus in an urban community in North-Western Nigeria. Pan African Medical Journal. 2018;29(1):1-7.
28. Wondemagegn AT, Bizuayehu HM, Abie DD, Ayalneh GM, Tiruye TY, Tessema MT. Undiagnosed diabetes mellitus and related factors in East Gojjam (NW Ethiopia) in 2016: a community-based study. Journal of public health research. 2017;6(1).

29. Tesfaye T, Shikur B, Shimels T, Firdu N. Prevalence and factors associated with diabetes mellitus and impaired fasting glucose level among members of federal police commission residing in Addis Ababa, Ethiopia. BMC endocrine disorders. 2016;16(1):68.
30. Abebe SM, Berhane Y, Worku A, Assefa A. Diabetes mellitus in North West Ethiopia: a community based study. BMC public health. 2014;14(1):97.
31. Mayega RW, Guwatudde D, Makumbi F, Nakwagala FN, Peterson S, Tomson G, et al. Diabetes and pre-diabetes among persons aged 35 to 60 years in eastern Uganda: prevalence and associated factors. PloS one. 2013;8(8):e72554.
32. Chege MP. Risk factors for type 2 diabetes mellitus among patients attending a rural Kenyan hospital. African journal of primary health care & family medicine. 2010;2(1).
33. Nyenwe EA, Odia OJ, Ihekwaba AE, Ojule A, Babatunde S. Type 2 diabetes in adult Nigerians: a study of its prevalence and risk factors in Port Harcourt, Nigeria. Diabetes research and clinical practice. 2003;62(3):177-85.
